# Supplementary material for: VEGF/VEGFR2 signaling regulates hippocampal axon branching during development
Source: eLife. 2019 Dec 23;8:e49818. doi: 10.7554/eLife.49818 (PMC6927742; doi:10.7554/eLife.49818)
Supplement: Transparent reporting form [file elife-49818-transrepform.docx]

***eLife’s* transparent reporting form**

We encourage authors to provide detailed information *within their submission* to facilitate the interpretation and replication of experiments. Authors can upload supporting documentation to indicate the use of appropriate reporting guidelines for health-related research (see [EQUATOR Network](http://www.equator-network.org/%20)), life science research (see the [BioSharing Information Resource](https://biosharing.org/)), or the [ARRIVE guidelines](http://www.plosbiology.org/article/info:doi/10.1371/journal.pbio.1000412) for reporting work involving animal research. Where applicable, authors should refer to any relevant reporting standards documents in this form.

If you have any questions, please consult our Journal Policies and/or contact us: [editorial@elifesciences.org](mailto:editorial@elifesciences.org).

**Sample-size estimation**

- You should state whether an appropriate sample size was computed when the study was being designed
- You should state the statistical method of sample size computation and any required assumptions
- If no explicit power analysis was used, you should describe how you decided what sample (replicate) size (number) to use

Please outline where this information can be found within the submission (e.g., sections or figure legends), or explain why this information doesn’t apply to your submission:

Detailed information of sample size can be found in the figure legends.

For *in vivo* experiments, the animal number was estimated as needed for obtaining the ethical animal permission, using an online software (<http://www.biomath.info/power/index.htm>; <http://isogenic.info/html/power_analysis.html>).

For *in vitro* experiments, a priori no power analysis was used to determine the sample size. Sample size was determined based on previous literature describing experiments of a similar nature.

**Replicates**

- You should report how often each experiment was performed
- You should include a definition of biological versus technical replication
- The data obtained should be provided and sufficient information should be provided to indicate the number of independent biological and/or technical replicates
- If you encountered any outliers, you should describe how these were handled
- Criteria for exclusion/inclusion of data should be clearly stated
- High-throughput sequence data should be uploaded before submission, with a private link for reviewers provided (these are available from both GEO and ArrayExpress)

Please outline where this information can be found within the submission (e.g., sections or figure legends), or explain why this information doesn’t apply to your submission:

Data regarding the replication of the experiments is provided in the respective figure legends. The number of independent experiments stated in the figure legends refers to biological replicates. Whenever a technical replicate is used, it is also stated in the figure legends. For *in vitro* experiments, a biological replicate is defined as an independent isolation of neurons from a different mouse litter.

Outlier testing was performed using the GraphPad Prism test for outliers. If an outlier was detected (p<0.05), the value was omitted from the analysis.

**Statistical reporting**

- Statistical analysis methods should be described and justified
- Raw data should be presented in figures whenever informative to do so (typically when N per group is less than 10)
- For each experiment, you should identify the statistical tests used, exact values of N, definitions of center, methods of multiple test correction, and dispersion and precision measures (e.g., mean, median, SD, SEM, confidence intervals; and, for the major substantive results, a measure of effect size (e.g., Pearson's r, Cohen's d)
- Report exact p-values wherever possible alongside the summary statistics and 95% confidence intervals. These should be reported for all key questions and not only when the p-value is less than 0.05.

Please outline where this information can be found within the submission (e.g., sections or figure legends), or explain why this information doesn’t apply to your submission:

Statistical analysis methods and N number can be found in figure legends. Raw data plots are mainly not presented in the Figures as the N number per group is always higher than 10. Exception are the graphs in Figures 5E and Supplementary Figure 5E in which the N number is smaller than 10 (as indicated also in the respective figure legends). The exact p-values are reported in the source data.

(For large datasets, or papers with a very large number of statistical tests, you may upload a single table file with tests, Ns, etc., with reference to sections in the manuscript.)

**Group allocation**

- Indicate how samples were allocated into experimental groups (in the case of clinical studies, please specify allocation to treatment method); if randomization was used, please also state if restricted randomization was applied
- Indicate if masking was used during group allocation, data collection and/or data analysis

Please outline where this information can be found within the submission (e.g., sections or figure legends), or explain why this information doesn’t apply to your submission:

For *in vivo* experiments, analysis of phenotype was done blind and unblinded after analysis was finished. Subsequently, mouse embryos were grouped by genotype.

**Additional data files (“source data”)**

- We encourage you to upload relevant additional data files, such as numerical data that are represented as a graph in a figure, or as a summary table
- Where provided, these should be in the most useful format, and they can be uploaded as “Source data” files linked to a main figure or table
- Include model definition files including the full list of parameters used
- Include code used for data analysis (e.g., R, MatLab)
- Avoid stating that data files are “available upon request”

Please indicate the figures or tables for which source data files have been provided:

An excel file including all detailed statistical information of the data presented in the study is provided as a source data file.
